# *Pediatric Neurology Briefs*: Year in Review

**DOI:** 10.15844/pedneurbriefs-31-1-1

**Published:** 2017-01

**Authors:** John J. Millichap

**Affiliations:** 1 Division of Neurology, Ann & Robert H. Lurie Children’s Hospital of Chicago, Chicago, IL; and Departments of Pediatrics and Neurology, Northwestern University Feinberg School of Medicine, Chicago, IL

**Keywords:** Neurology, Pediatrics, Child Development, Nervous System Diseases, Brain Diseases

## Abstract

*Pediatric Neurology Briefs* (PNB) has been published monthly since 1987 as a continuing education service designed to expedite and facilitate review of current medical literature concerning pediatric neurology.

*Pediatric Neurology Briefs* (PNB) has been published monthly since 1987 as a continuing education service designed to expedite and facilitate review of current medical literature concerning pediatric neurology. PNB has reviewed and referenced articles from over 200 journals since inception. So far, PNB consists of 30 volumes with greater than 3700 articles, and includes over 10,000 citations referencing the works of approximately 28,000 scholarly authors. PNB was relaunched as an open access, peer-reviewed, journal with an expanded editorial board in January 2015. The Editors of PNB select source articles using criteria that include recent publication in a peer-reviewed journal and a topic of clinical value to practicing pediatric neurologists. Contributing Editors provide detailed summaries of published articles, followed by commentaries based on their experience and corroborated by appropriate supplementary citations. Also in 2015, PNB launched a new website and content management system capable of organizing peer-review and providing improved indexing, DOI assignment, and online full-text article view. Prior to the internet, the Editor periodically compiled and published the PNB articles in book form with index, according to subject heading and in chronological order [1-3].

In 2016, digitization of the 30 years of back issues was completed. There are now over 3700 full text open access articles available on the journal website. Also in 2016, PNB was selected for inclusion in PubMed Central® (PMC). PMC is a free archive of biomedical and life sciences journal literature at the U.S. National Institutes of Health's National Library of Medicine (NIH/NLM). The two most highly accessed articles published in 2016 covered topics related to neuroinfectious disease including Zika virus [4] and acute myelitis [5].

The Editors would like to take this opportunity to sincerely thank the 2016 Contributing Editors for their effort and expertise (listed below in alphabetical order):

José Biller, MD

Chelsea Chambers, MS, CGC

Melissa G. Chung, MD

Radhika Dhamija, MD

Deborah Gaebler-Spira, MD

Amy A. Gelfand, MD, MAS

Cecil D. Hahn, MD, MPH

Chafic Karam, MD

Marisa Klein-Gitelman, MD

Lawrence J. Jennings, MD, PhD

Csaba Juhász, MD, PhD

Anthony P. Kontos, PhD

Cynthia R. LaBella, MD

Aleksandra Mineyko, MD

Leena B. Mithal, MD, MSCI

Sakkubai Naidu, MD

Srishti Nangia, MD

Dragos A. Nita, MD, PhD

Katrin Õunap, MD, PhD

Andrea C. Pardo, MD

Andrea Poretti, MD

Colin Quinn, MD

Vamshi K. Rao, MD

David G. Ritacco, MD, PhD

Maura Ryan, MD

Lalitha Sivaswamy, MD

Rochelle Sweis, DO

Martin Tisdall, FRCS, MD

Molly Tracy, MD

Sophia Varadkar, MRCPI, PhD

Michelle M. Yee, CPNP

The mission of PNB remains the same: “*Pediatric Neurology Briefs* is a continuing education service designed to expedite and facilitate the review of current scientific research and advances in child neurology and related subjects.” The Editors of PNB endeavor to deliver a ‘clinical pearl’ with each commentary that will be of interest and use in clinical practice. Topic experts interested in contributing to PNB as authors or reviewers are invited to contact the Editor at j-millichap@northwestern.edu.
